# Coding Gene SNP Mapping Reveals QTL Linked to Growth and Stress Response in Brook Charr (*Salvelinus fontinalis*)

**DOI:** 10.1534/g3.112.001990

**Published:** 2012-06-01

**Authors:** Christopher Sauvage, Marie Vagner, Nicolas Derôme, Céline Audet, Louis Bernatchez

**Affiliations:** * Département de Biologie, Institut de Biologie Intégrative et des Systèmes (IBIS), Université Laval, Québec (Québec) Canada, G1V 0A6; † INRA, UR1052, Unité de Génétique et d’Amélioration des Fruits et Légumes, 84143 Montfavet, France; ‡ Institut des sciences de la mer de Rimouski (ISMER), Université du Québec à Rimouski, Rimouski (Québec) Canada, G5L 3A1; § Institut du Littoral et de l’Environnement, LIENSs, UMR7266, 17000 La Rochelle, France

**Keywords:** linkage mapping, QTL detection, single-nucleotide polymorphism, growth, stress response, *Salvelinus fontinalis*

## Abstract

Growth performance and reduced stress response are traits of major interest in fish production. Growth and stress-related quantitative trait loci (QTL) have been already identified in several salmonid species, but little effort has been devoted to charrs (genus *Salvelinus*). Moreover, most QTL studies to date focused on one or very few traits, and little investigation has been devoted to QTL identification for gene expression. Here, our objective was to identify QTL for 27 phenotypes related to growth and stress responses in brook charr (*Salvelinus fontinalis*), which is one of the most economically important freshwater aquaculture species in Canada. Phenotypes included 12 growth parameters, six blood and plasma variables, three hepatic variables, and one plasma hormone level as well as the relative expression measurements of five genes of interest linked to growth regulation. QTL analysis relied on a linkage map recently built from *S. fontinalis* consisting of both single-nucleotide polymorphism (SNP, *n* = 266) and microsatellite (*n* =81) markers in an F_2_ interstrain hybrid population (*n* = 171). We identified 63 growth-related QTL and four stress-related QTL across 18 of the 40 linkage groups of the brook charr linkage map. Percent variance explained, confidence interval, and allelic QTL effects also were investigated to provide insight into the genetic architecture of growth- and stress-related QTL. QTL related to growth performance and stress response that were identified could be classified into two groups: (1) a group composed of the numerous, small-effect QTL associated with some traits related to growth (*i.e.*, weight) that may be under the control of a large number of genes or pleiotropic genes, and (2) a group of less numerous QTL associated with growth (*i.e.*, gene expression) and with stress-related QTL that display a larger effect, suggesting that these QTL are under the control of a limited number of genes of major effect. This study represents a first step toward the identification of genes potentially linked to phenotypic variation of growth and stress response in brook charr. The ultimate goal is to provide new tools for developing Molecular Assisted Selection for this species.

Quantitative trait loci (QTL), the portions of a species’ genome that affect the variation of heritable phenotypic traits, are revealed by the association of phenotypes with molecular markers. They provide insight into the number of loci affecting a trait and on the distribution of the QTL effects of each locus (Lynch and Walsh 1998). QTL analysis has become a popular method for studying the genetic basis of continuous variation in a variety of systems and is now an integral tool in medical genetics, livestock production, plant breeding, and population genetics of model organisms (Slate 2005). Thus, this approach is of great interest when studying experimental populations obtained from livestock species. Knowledge gained from these analyses can be used to improve traits of economic importance such as growth, resistance to pathogens, sexual maturation, or stress response through the clarification of their underlying genetic basis. Such information helps the development of selection programs aiming to improve efficiency, yield, and production sustainability (Haley and Koning 2006). Indeed, the use of QTL in marker-assisted selection for phenotypic traits of interests offers great potential and provides the basis for cloning genes underlying the genetic architecture of these traits (Mackay 2001; Remington *et al.* 2001). This approach has been applied in many livestock species but was introduced relatively recently (a decade ago) in farmed aquatic species such as rainbow trout *Oncorhynchus mykiss* (Ozaki *et al.* 2001), Atlantic salmon *Salmo salar* (Houston *et al.* 2008), and Pacific oyster *Crassostrea gigas* (Sauvage *et al.* 2010a) to improve disease resistance or growth. QTL studies provide a framework for the identification of genes and genetic architecture underlying heritable variation within populations and divergence among them. However, this has not proven true from QTL studies alone, which need to be supported by candidate genes approach to fully detect and understand the complex traits architecture (for review, see Rockman 2011).

Growth is one of the most important fitness traits targeted toward a more efficient production of livestock species. The variation of this complex trait relies on a network of genes (*e.g.*, pleiotropy) and on many surrounding environmental inputs (Wang *et al.* 2002), such as seasonal variations of environmental conditions (Makinen and Ruohonen 1992), food availability (Ali *et al.* 2003; Bureau *et al.* 2006), competition (Metcalfe 1986; Blanchet *et al.* 2007), and other biotic and abiotic factors (*e.g.*, thermal tolerance) (Jackson *et al.* 1998). Moreover, growth is known to be correlated with variations of other life-history traits, such as gonad maturation processes and reproductive timing (Schaffer 1979; Thorpe 1994; Devlin and Nagahama 2002). Despite the numerous factors influencing growth, in most studies in which investigated its heritability revealed moderate-to-high levels of heritability throughout a wide range of taxa (Wringe *et al.* 2010).

Stress response, which has been defined as a “diversion of metabolic energy from animal’s normal activities” (Barton and Schreck 1987), is another important fitness-related trait in aquaculture production. In aquaculture facilities, fish are submitted to many stressful manipulations (handling, sorting, transportation, vaccination). All these have the potential to initiate a severe stress response (Barton and Iwama 1991; Portz *et al.* 2006), which can affect other relevant production traits, including growth performance, feed conversion, immunocompetence, reproductive performance, and disease resistance (Pickering 1981; Adams 1990; Pottinger and Pickering 1997; Wendelaar Bonga 1997; Iversen *et al.* 1998; Barton 2002).

Salmonids are the most important farmed fish group in Canada. As is the case for other livestock, their growth performance and stress response are of particular economical interest. The mapping of QTL associated with growth traits has been extensively documented in several salmonid species, including rainbow trout (Martyniuk *et al.* 2003; O’Malley *et al.* 2003; Perry *et al.* 2005; Drew *et al.* 2007; Moghadam *et al.* 2007a; Wringe *et al.* 2010), coho salmon *Oncorhynchus kisutch* (McClelland and Naish 2010), Arctic charr *Salvelinus alpinus* (Moghadam *et al.* 2007b), Atlantic salmon (Reid *et al.* 2005), and chinook salmon *Oncorhynchus tshawyscha* (Du *et al.* 1993). The results of these studies have provided insight into the genomic architecture of growth-regulating regions within the salmonid genome. For example, homologous linkage groups with similar QTL effects on fork length and body weight have been observed among different species (O’Malley *et al.* 2003; Drew *et al.* 2007; Moghadam *et al.* 2007b; Wringe *et al.* 2010). It has also been demonstrated that duplicate copies of growth hormone coding sequences are located in the homologous linkage groups RT-2/9 and that genetic markers close to these regions have been identified as body weight QTL regions in both rainbow trout and Arctic charr (Moghadam *et al.* 2007b). In addition, recent studies have reported the identification of QTL and candidate genes related to plasma cortisol concentration in rainbow trout (Drew *et al.* 2007; Vallejo *et al.* 2009) as well as three potential QTL related to stress response in sea bass *Dicentrarchus labrax* (Massault *et al.* 2010). Despite these studies, QTL related to stress response remain poorly studied in fish.

Using brook charr (*Salvelinus fontinalis*), one of the most economically important freshwater aquaculture species in Canada, we aimed to extend the work on salmonids by the identification of QTL underlying two phenotypic traits highly relevant to aquaculture production, *i.e.*, growth performance and stress response. Our analyses were based on a single-nucleotide polymorphism (SNP)-based consensus linkage map (Sauvage *et al.* 2012) identified by RNA-seq and thus all located in coding genes and a set of 27 traits related to growth and stress response that were phenotyped in 171 F_2_ full-sib individuals. These phenotypes included measurements on 12 growth parameters, six blood and plasma variables, three hepatic variables, one stress hormone plasma level, and the expression of five genes of interest related to growth. This study represents a first step toward the identification of genes potentially linked to phenotypic variation of growth and stress response in brook charr. The ultimate goal is to provide new tools for developing molecular-assisted selection for this species.

## Materials and Methods

### Biological material and fish crosses

The F_2_ population used in this study was obtained from a cross between a domestic population (D) that has been used in aquaculture in Québec (Canada) for more than 100 years and another one (L) that was derived from an anadromous population originating from the Laval River near Forestville (north of the St. Lawrence River, QC, Canada; see Castric and Bernatchez 2003). In previous studies investigators showed that these two strains are highly genetically distinct on the basis of both on gene expression analyses (Bougas *et al.* 2010) and Fst (The fixation index, Fst is a measure of population differentiation) estimate of 0.187 (± 0.009) on the basis of microsatellite data (Martin *et al.* 1997). Breeders from the L population were kept in captivity for three generations at the Station aquicole de l’Institut des Sciences de la Mer (ISMER, Pointe-au-Père, QC, Canada, 48°31′N, 68°28′W), whereas those from the D population were obtained from Pisciculture de la Jacques Cartier (Cap-Santé, QC, Canada). In 2005, 10 sires of each population (L and D; F_0_ generation) were crossed with 10 dams (L and D) to generate 10 full-sib outbred hybrid (LD) crosses (F_1_ generation). The biparental cross of six F_1_ individuals then resulted in three F_2_ families. At each stage, families were kept separately at the Laboratoire Régional des Sciences Aquatiques (LARSA, Université Laval, QC, Canada) under identical controlled conditions of temperature and photoperiod. Fertilized eggs were incubated at 6°. After hatching, progeny were kept at 8° with a 12L:12D photoperiod. From the three F_2_ families, a single F_2_ family was used in the present study and chosen according to its lower mortality rate to minimize bias (*e.g.*, segregation distortion) in the subsequent analyses.

### Fish-rearing conditions

Fish from the F_2_ hybrid population hatched in January 2008 and were reared in a single indoor flow-through circular tank in fresh water at the Station aquicole de l’ISMER under natural photoperiod and temperature conditions. All fish were tagged with electronic PIT-tags at 5 months for individual identification at all sampling points. Density was maintained below 35 kg of fish per m^3^. Fish were fed daily with commercial pellets according to fish age and water temperature (from 1.5 to 5.3% of body weight).

### Genetic linkage map

Marker development and construction of the genetic map used for the QTL analysis of this study have been presented in details in a companion study (Sauvage *et al.* 2012). In brief, a normalized cDNA library was sequenced on a GS-FLX 454 Titanium sequencer. Assembled contigs were screened for SNPs using the implemented tool in CLC Genomic Workbench v3.7 based on Brockman *et al.* (2008). A subset of 300 SNP markers in the mapping population was validated from the whole set of potential SNP detected in the contigs using a four step validation approach detailed in Sauvage *et al.* (2012). In addition to SNP markers that were genotyped on F2 progeny, a total of 81 microsatellites markers available in the literature were also used to build the map.

### Data collection

#### Sampling and phenotyping for growth:

Samplings were performed on 171 F_2_ progeny of age 1+ on four occasions: T1: May 2009 (*n* = 171; 72.6 ± 1.5 g), T2: July 2009 (*n* = 171; 145.8 ± 2.5 g), T3: August 2009 (*n* = 85; 223.9 ± 5.5 g), and T4: November 2009 (*n* = 86; 273.7 ± 6.4 g). Fish had fasted for 12 hr before each sampling. Fish were captured and immediately placed in an anesthetic solution (3-aminobenzoic-ethyl-ester-acid MS-222, 0.16 g L^-1^) with constant aeration. They were identified using a PIT-tag reader, measured (± 0.1 cm), and weighed (± 0.1 g).

Two growth-related parameters were calculated for each period:

(1) $$\text{Specific}\;\text{growth}\;\text{rate}\left(\text{SGR}\right)\;\text{=}\;\text{100}\;\times \;\left(\text{ln}\;{\text{w}}_{\text{final}}\;-\;\text{ln}\;{\text{w}}_{\text{initial}}\right){\text{day}}^{\text{-1}}$$

where SGR is % day^−1^ and w_initial_ and w_final_ are the initial and final mean body weights for each period, respectively; and

(2) $$\text{Fulton}\;\text{index}\;\text{=}\;\text{100}\;\times \;\left[{\left(\text{L}/\text{w}\right)}^{3}\right]$$

where L is fish length and w is fish weight.

Blood from the 85 anesthetized fish randomly sampled in August 2009 was sampled by caudal puncture using cooled 1-mL heparinized syringes. Blood samples were used for hematocrit, plasma cortisol, plasma osmolality, and plasma chloride measurements. Fish were killed by decapitation immediately after caudal puncture; this was done according to Canadian Council of Animal Protection recommendations and protocols approved by the University Animal Care Committee. All manipulations were performed quickly so that blood samples were obtained within 2 to 3 min of transfer into the anesthetic solution. Blood samples were immediately centrifuged at 5000 rpm (8500g) for 5 min and collected plasma was immediately frozen at −80° until analyses. Hematocrit (percentage of red blood cells in the centrifuged blood volume) was measured in duplicate in capillary tubes centrifuged for 3 min at 4000 rpm (6800g). Fish were dissected on ice (4°) to collect the whole liver without the gall bladder because it affects RNAs integrity. Livers were weighed, immediately frozen in liquid nitrogen, and stored at −80° for subsequent gene expression measurements. The hepatosomatic index (HSI) was determined for all fish by use of the following relationship:

(3) $$\text{HSI}\;\text{=}\;\left({\text{w}}_{\text{liver}}\;\times \;\text{100}\right)/\left({\text{w}}_{\text{fish}}\;-\;{\text{w}}_{\text{liver}}\right)$$

where w_liver_ is the liver weight and w_fish_ is the fish weight.

##### Quantitative analyses of gene expression:

The relative expression of five genes involved in growth metabolism were measured in liver tissue from the 85 fish sampled in August 2009; these genes were *growth hormone receptor* (*ghr*), *insulin growth factor-1* (*igf1*), *insulin growth factor-1 receptor* (*igf1r*), *elongation factor-1* (*ef1*), and *β-actin*. The gene encoding the *18s* ribosomal unit was used as a reference gene because its expression was constant between samples. Total RNA was extracted from 30 mg of liver from each fish using RNeasy Plus Mini Kit (QIAGEN, Inc., ON, Canada). Total RNA integrity and quantity were determined using a Nanodrop spectrophotometer (Nanodrop ND-1000 v3.3.0, NanoDrop Technologies, Inc., Wilmington, DE) and a 1.2% agarose gel. To obtain cDNAs, reverse transcription was performed on 1 µg of total RNA in duplicate for each sample using a Quantitect Reverse Transcription kit with integrated removal of genomic DNA contamination (QIAGEN, Inc.). cDNA integrity and quantity were also checked using Nanodrop spectrophotometer. Duplicate cDNAs were then pooled for each sample and real-time polymerase chain reaction (PCR) was performed using the iCycler iQ (Bio-Rad Laboratories Inc., ON, Canada).

The *igf1* and *ghr* primers were designed specifically for *S. fontinalis* by Côté *et al.* (2007) using sequence information from *Oncorhynchus kisutch* [GenBank:AF403539] and *O. keta* [GenBank:AF063216]. The mRNA sequences for *igf1r*, *ef1*, *β-actin*, and *18s* were not available for *S. fontinalis* in the GenBank databases. Consequently, we designed primers from closely related salmonids species by using Primer 3 software (Rozen and Skaletsky 2000) to obtain PCR products ranging from 90 to 150 bp. We used mRNA *igf1r* sequences from *O. mykiss* [GenBank:AF062496]; alignment between mRNA *β-actin* sequences from *O. mykiss* [GenBank:AF157514] and *S. salar* [GenBank:NM_001123525]; mRNA *ef1* sequences from *O. tshawytscha* [GenBank:FJ890356]; and mRNA *18s* sequence from *S. salar* [GenBank:AJ427629]. Sequences of primers used are summarized in Table 1.

**Table 1:** Primers used for gene expression analysis by RT-PCR

Target Gene	Primer Set (5′→3′)
*ghr*	Forward: CCCACTGCCCCCTGTATCT
	Reverse: CTTCAGAAGGAGGCTGTTTTGC
*β-actin*	Forward: GCTGTCTTCCCCTCCATCGT
	Reverse: TCTCCCACGTAGCTGTCTTTCTG
*igf1*	Forward: CAGGCATCCAGATTGTGCAA
	Reverse: ACCATGTTCTGAGAATTCCTGTGTT
*igf1r*	Forward: AGACCCAGTTTCTGAATTTCACC
	Reverse: GTTCTTATAAGGCGCCTCTTTGT
*ef1*	Forward: GCCCCTCCAGGATGTCTACA
	Reverse: ACGGCCCACGGGTACTG
*18s*	Forward: CCCCGTAATTGGAATGAGTACACTTT
	Reverse: ACGCTATTGGAGCTGGAATTACC

For details, see *Materials and Methods*. RT-PCR, reverse transcription polymerase chain reaction; *ghr*, growth hormone receptor; *β-actin*, b**e**ta actin; *igf1*, insulin growth factor-1; *igf1r*, insulin growth factor-1 receptor; *ef1*, elongation factor-1; *18s*, 18s ribosomal subunit.

Amplicons were sequenced to check the specificity of forward and reverse primers: ligation was performed with the TOPO TA Cloning Kit for Sequencing (Invitrogen Inc., ON, Canada) and then transformation was performed using One Shot Chemically Competent *E. coli* (Invitrogen Inc.). Bacterial cDNA was extracted using EZNA Plasmid Mini Kit I (Omega Bio-Tek, Norcross, GA). Nucleotides were isolated with the Ultra-Step Dye Terminator Removal Kit (Eazy Nucleic Isolation; Ezna, Omega Bio-Tek) and sequenced in forward and reverse sense using the Big Dye Terminator v3 chemistry (Applied Biosystems, Foster City, CA). Alignment between the sequence obtained and the sequence used for primer design was performed for each gene; the similarity percentages obtained were 100% for *18s*, 98% for *β-actin*, 94% for *ef1*, and 97% for *igf1r*.

Real-time PCR analyses for each gene were performed in duplicate for each pool of cDNA in a total volume of 15 µL containing 5 µL of cDNA (dilution: 10^−2^), 0.5 µL of primers (10 µmol L^-1^), and 7.5 µL of 2X iQ SYBR Green Supermix (Bio-Rad Laboratories, Inc.). Thermal cycling of real-time PCR was initiated with incubation at 95° for 13.5 min for activation of the hot-start enzyme, iTaq DNA polymerase. After this initial step, 45 cycles of PCR were performed. Each PCR cycle consisted of heating at 95° for 30 s for denaturing, at 60° for 1 min for annealing, and at 72° for 30 s for extension. Cycle threshold (CT) values corresponded to the number of cycles at which the fluorescence emission monitored in real time exceeded the threshold limit. CT values were automatically calculated on the log curve for each gene. For each plate, a melt curve was established to ensure the presence of a unique amplicon. Thus, the 45 cycles for cDNA amplification were followed by one cycle at 95° for 1 min, one cycle at 55° for 1 min, and 80 cycles at 55° for 10 sec. Standard curves were established in triplicate for each gene by plotting the CT values against log_10_ of five different dilutions (in triplicate) of a pool of all cDNA sample solutions. Real-time PCR efficiency (E) was determined for each gene from the slope of the mean standard curve according to the equation (4):

(4) $$\text{E}\;\text{=}\;{\text{10}}^{\left[\text{-1}/\text{slope}\right]}$$

To determine the relative quantity of target gene-specific transcripts present in the different samples, relative expression ratios were calculated according to equation (5). The relative expression ratio for a considered gene is based on the PCR efficiency (E) and the CT of a sample *vs.* the control (standard group); it is expressed in comparison to the reference gene (*18s*) according to Pfaffl’s equation (Pfaffl 2001):

(5) $$\text{Ratio}\;=\;\left[{\left({\text{E}}_{gene}\right)}^{\Delta \text{CT}gene\left(\text{control-sample}\right)}\right]/\left[{\left({\text{E}}_{\mathrm{18}s}\right)}^{\Delta \text{CT}\mathrm{18}s\left(\text{control-sample}\right)}\right]$$

The mean CT of samples from standard curves and diluted at 10^−2^ was used as the standard group because it was the most representative cDNA of the population tested. Normalization was used to correct for intensity distortions as well as spatial variation in signal level across the different real-time PCR runs.

##### Physiological measurements:

Plasma glucose concentrations and hepatic glycogen levels were measured in all 85 fish sampled in August 2009. Plasma glucose concentrations (mg ⋅ mL plasma^-1^) were measured using a commercial kit (QuantiChrom Glucose Assay kit; BioAssay Systems, Hayward, CA). Hepatic glycogen levels (mg ⋅ g of liver^-1^) were assessed using the amyloglucosidase digestion method (Carr and Neff 1984) followed by glucose concentration determinations (QuantiChrom Glucose Assay kit; BioAssay Systems). All measurements were done in duplicates, which all varied by less than 5% (data not shown).

##### Sampling and phenotyping for stress:

In November 2009, the baseline of the stress response for 86 F_2_ progeny was determined. Water level was gradually lowered in the single circular tank, and fish were individually captured without being pursued. They were individually anesthetized (MS-222; 0.16 g L^-1^), identified using a PIT-tag reader, and then blood was sampled (0.3 mL) by caudal puncture using cooled heparinized syringes. After a full recovery, fish were returned to their initial tank.

One week later, the stress experiment was performed on the 86 fish. Water level was again gradually lowered in the single circular tank, and fish were individually captured without being pursued. After being captured, each fish was individually stressed by one minute of handling out of water in a small net. After the handling stress, fish were placed in groups of six in smaller opaque tanks (volume: 80 L; fish density: 1.95 10^−3^ kg L^-1^) until blood sampling. Three hours after the handling stress (Bastien 2010), fish were anesthetized (MS-222; 0.16 g L^-1^). Fish were identified using a PIT-tag reader, weighed (± 0.1 g), and measured (± 0.1 cm) before caudal puncture. Fish were killed by decapitation immediately after caudal puncture; this was done according to Canadian Council of Animal Protection recommendations and protocols approved by the University Animal Care Committee. Sex and maturation status were visually determined by inspection of the gonads. Fish were classified as mature (when eggs and sperm could be collected by stripping) or immature.

All samplings (before and after acute stress) were done between 1:00 pm and 2:00 pm to avoid circadian variation effects on plasma cortisol concentration (Audet and Claireaux 1992). All manipulations were done quickly so that blood was obtained within 2 to 3 min after transfer to the anesthetic solution. Plasma was obtained by centrifugation (5 min, 5000 rpm; 8500g) and then stored at −80° until analysis.

##### Physiological measurements of stress response:

Plasma cortisol concentrations (µg dL^-1^ of plasma) were measured for each fish using a commercial radioimmunoassay kit (ImmuChem Cortisol ^125^I RIA kit; MP Biomedicals, Cleveland, OH) validated in fish (Vijayan and Moon 1992). Radioactivity of the [^125^I]-labeled cortisol tracer was quantified using the automatic CliniGamma 1272 gamma counter (LKB-Wallac, Wallac, Finland). For each assay, a standard curve was constructed and cortisol levels were within the linear range of the assay. All measurements were done in duplicate, and replicate measurements varied by less than 5%.

Plasma osmolality (mmol kg^-1^) was measured using a Vapro Vapor Pressure Osmometer 5520 (Wescor Inc., Logan, UT) and plasma chloride (mmol L^-1^) was measured using the Chloride Analyzer 925 (Corning Medical and Scientific, England). All measurements were performed in duplicate, and replicate measurements varied by less than 5%.

##### Phenotype data analyses:

Because some traits were measured at several sampling times (*i.e.*, size and weight were measured at T1, T2, and T3 on 85 of the F_2_ progeny and again at T4 on the remaining 86 fish), we decided to perform the QTL analyses on the largest time interval (from T1 to T3 for the first 85 sampled fish and from T1 to T4 for the last 86 sampled fish) to maximize QTL detection and variations in phenotype measurements. QTL detection related to size, weight, SGR, and Fulton index were then performed with the data set obtained at T4 (November), whereas the QTL detection for traits related to blood parameters (hematocrit, plasma chloride, plasma osmolality, and plasma glucose), gene expression (*ghr*, *igf1*, *igf1R*, *ef1*, and *β-actin*), and liver variables (liver fresh weight, hepato-somatic index, and hepatic glycogen) were performed on the data set obtained at T3 (August).

Correlations within phenotypic traits related to growth performance (collected at T1, T2, T3, and T4) and stress response (collected at T4) were determined by correlation matrix using Statistica 6 for Windows (StatSoft Inc., Tulsa, OK). Normality was tested on residuals using Kolmogorov-Smirnov test. Phenotypic traits that were not normally distributed were log_10_ transformed. Another assumption for correlation matrix is the absence of outliers. Thus, outliers (*n* = 10) were identified and removed from the database using the scatterplot box-plot function (Statistica). Differences were considered significant at *P* < 0.05. The graphical correlation matrix displaying the positive and negative correlations between the phenotypes related to growth and the phenotypes related to the stress response were obtained using the R package “Corrplot” (Friendly 2002).

#### QTL detection:

QTL analyses for the aforementioned 27 phenotypic traits were performed using the [R] package R/qtl (v. 1.18-7, August 2010, http://www.rqtl.org/) (Broman *et al.* 2003) only on the sex-averaged (consensus) linkage map. The following approach was used: (1) A single QTL analysis was performed using the Haley-Knott (HK) regression method (10 000 permutations) (Haley and Knott 1992) to reveal which linkage groups (LGs) were carrying QTL. The most probable position of the QTL was defined at the position giving the largest log_10_ of the odd ratio (LOD) score; this QTL was fixed. (2) Then, a full model that incorporates all those loci identified in the single QTL scan was used to refine the positions and to estimate effects and PVE across the genome with a resolution of 5 cM. (3) Finally, the model best fitting our data were used to compute the percent variance explained (PVE) associated with the QTL. The chromosome-wide and genome-wide thresholds were calculated for each LG using 10,000 permutations. The 1.5 LOD confidence intervals were determined for all analyses following the Bayesian method implemented in the “bayesint” function in R/qtl. The bayesint function calculates an approximate interval (end points around the maximum LOD) for a given chromosome using the genome scan output. Allele effects were determined using the effect plot function in R/qtl with the QTL peak marker or the marker nearest to the peak as the reference marker. The additive effect was estimated as one-half of the difference between the two homozygous genotype values. The dominance effect was estimated as the deviation of the heterozygous genotype values from the average of the two homozygous genotypic values (Lynch and Walsh 1998). The resulting value indicated that the progeny should closely resemble one of the two parental lines rather than having an intermediate phenotype (Bennewitz and Meuwissen 2010).

## Results

### Genetic linkage map

The detailed building of the linkage map is described in Sauvage *et al.* (2012). Briefly, the dataset used to build the linkage map comprised 81 microsatellite markers and 256 SNP located in coding gene regions, for a total of 337 markers. Forty LGs were generated, which is close to the haploid number of chromosomes in brook charr (2n = 84). The consensus (sex-averaged) map contained 266 markers (191 SNPs and 75 microsatellites) displayed in the 40 LGs (see Table 2 in Sauvage *et al.* 2012 for details). The LG length ranged from 1.4 cM to 132 cM, for a total map length of 2047.5 cM. The average marker spacing per LG ranged from 0.7 to 21.3 cM and was estimated at 8.3 cM over the whole genome. The genome coverage was estimated at 89% as following: a complete female map is usually expected to be roughly 25 Morgans, assuming approximately 50 cM per chromosome arm pair (50 chromosome arm sets in the brook charr). In the present study, the female map that covers a total of 22.48 Morgans represents about 89% of the genome. The exact position and order of the 266 markers among the 40 LGs are given in supplementary Table 2 in Sauvage *et al.* (2012).

### Phenotyping

**Figure 1:**
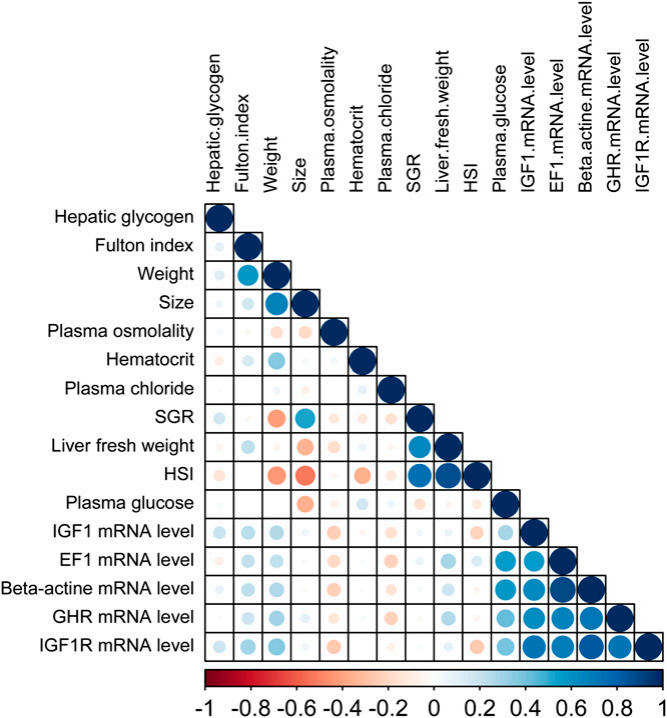
Correlation matrix displaying the positive (blue) and negative (red) correlations between the phenotypes related to growth measured at T3. Phenotypes have been clustered according to their correlation factor. The graphical representation was given by the R package “Corrplot” (Friendly 2002). SGR, specific growth rate; HSI, hepato-somatic index; *igf1*, *insulin growth factor-1*; *ef1*, *elongation factor-1*; *β-actin*, *beta -actin*; *ghr*, *growth hormone receptor*; *igf1r*, *insulin growth factor-1 receptor*. Color and size variation of circles in the figure are proportional level and direction (positive or negative) of the correlation between traits.

The detailed statistical descriptions of the 27 phenotypes analyzed are reported in Table 2 and Table 3. Among the phenotypes measured for growth related trait, standard deviations (SD) of the mean varied highly, from 0.07 for the Fulton’s condition factor at T3 up to 14.53 for hepatic glycogen. SD for SGR _T2-T3_ (0.24) was almost 2.5-fold greater than that for SGR _T1-T3_ (0.10). SD for stress-related phenotypes also varied highly, from 5.81 for the plasma chloride difference between before and after acute stress to 17.26 for plasma osmolality difference between, before, and after acute stress. The hierarchical clustering of phenotypes on the basis of correlation revealed two main clusters. The first is composed of six closely related phenotypes; size _T4_, weight _T4_, SGR _T1-T4_, Fulton’s condition factor _T4_, HSI _T3_, and liver weight _T3_ (Figure 1). The second cluster is composed of gene expression (mRNA levels of *ef1*, *igf1*, *igf1r*, *ghr*, and *β-actin* ; Figure 1). Phenotypes related to stress response and measured at T4 were expressed as the difference in levels before and after acute stress (response intensity). A negative correlation (*r* = −0.25; *P* = 0.021) was found between plasma chloride and plasma cortisol response intensity, while a positive correlation (*r* = +0.34; *P* = 0.001) was measured between plasma cortisol and plasma osmolality response intensities.

**Table 2:** Descriptive statistics of phenotypic traits related to growth measured in sampled fish

	Specific Growth Rate, % d^-1^				Length, cm			
	T1–T2	T2–T3	T1–T3	T1–T4	T1	T2	T3	T4
N	171	171	85	86	171	171	85	86
Min	−0.12	0.27	0.78	0.51	15.60	18.00	22.00	24.60
Max	1.53	2.16	1.20	0.89	23.40	31.60	31.60	32.90
Mean ± SD	1.16 ± 0.17	0.63 ± 0.24	0.98 ± 0.10	0.68 ± 0.08	19.14 ± 1.61	23.09 ± 1.92	27.04 ± 1.86	28.75 ± 1.75

	Fulton’s Condition Factor				Blood and Plasma Variables			
	T1	T2	T3	T4	Hematocrit, % of red cells	Chloride, mmol L^-1^	Osmolality, mmol kg^-1^	Glucose, mg mL^-1^
N	171	171	85	86	81	85	85	85
Min	0.62	0.37	0.97	0.82	28.00	111.93	301.50	0.35
Max	1.36	1.49	1.53	1.50	46.00	155.00	350.50	1.22
Mean ± SD	1.02 ± 0.12	1.18 ± 0.13	1.11 ± 0.07	1.14 ± 0.1	36.60 ± 3.72	133.58 ± 5.09	319.87 ± 10.21	0.67 ± 0.18

	Liver Fresh Weight, g	Hepato-somatic Index, HSI	Hepatic glycogen, mg g^-1^	mRNA Level in Liver (With Respect to *18s* mRNA Level)				
				*ghr*	*igf1*	*igf1r*	*ef1*	*β-actin*
N	171	171	85	83	83	83	82	83
Min	0.95	0.35	65.39	0.003	0.002	0.001	0.0003	0.002
Max	10.35	3.34	146.32	1.41	1.13	0.57	0.73	1.04
Mean ± SD	3.65 ± 1.86	1.57 ± 0.82	109.42 ± 14.53	0.16 ± 0.24	0.09 ± 0. 17	0.07 ± 0.10	0.07 ± 0.12	0.09 ± 0.15

N, number of observations, Min, minimum value obtained; Max, maximal value obtained, Mean ± SD: mean ± SD of the values measured. T1, May; T2, July; T3, August; T4, November; *18s*, 18s ribosomal subunit; *ghr*, *growth hormone receptor*; *igf1*, *insulin growth factor-1*; *igf1r*, *insulin growth factor-1 receptor*; *ef1*, *elongation factor-1*; *β-actin*, *beta-actin*.

**Table 3:** Descriptive statistics of phenotypic traits related to stress measured in the plasma of sampled fish in November 2009 (T4)

	Cortisol, µg dL^-1^	Osmolality, mmol kg^-1^	Chloride, mmol L^-1^
	Difference between before and after acute stress	Difference between before and after acute stress	Difference between before and after acute stress
N	86	86	86
Min	−8.39	−68.00	−15.00
Max	29.44	50.00	21.00
Mean ± SD	5.02 ± 6.63	3.16 ± 17.26	−1.47 ± 5.81

N, number of fish tested; Min, minimum value obtained; Max, maximal value obtained; Mean ± SD, mean ± SD of the values measured.

### QTL detection

#### Traits related to growth:

A total of 18 of the 40 LGs comprised QTL related to one of the phenotypic traits. For growth, 63 QTL were identified across the 22 growth-related phenotypes, with 8 QTL at the genome-wide level of significance and 55 at the chromosome-wide level. The greatest number of QTLs identified for a single trait was estimated at 12 for individual body weight (T4) and specific growth rate (SGR _T1-T4_), whereas the lowest number was estimated at one for blood hematocrit, plasma osmolality, and plasma glucose phenotypes as well as for the expression of *igf1*, *igf1r*, and *ghr* genes. LG22 carried the greatest number of QTL, with a total of six QTL related to six different phenotypes (size _T4_, weight _T4_, SGR _T1-T3_, SGR _T1-T4_, HSI _T3_, and liver weight _T3_). The minimal, maximal, and average LOD scores associated with the 63 QTL were estimated at 3.01 (QTL for Fulton index T4), 15.99 (QTL for HSI T3), and 8.29, respectively (Table 4). The respective minimal, maximal, and average PVE by the identified QTL related to growth were estimated at 3.08% (QTL linked to SGR _T1-T4_ on LG 3 at 44.8 cM), 17.27% (QTL linked to hematocrit on LG 4), and 6.49%, respectively (Table 4). Some QTL linked to different traits were colocalized on a same linkage group. Thus, QTL related to size, weight, and SGR were colocalized on LGs 3, 12, 20, 22, 24, and 32 and QTL linked to HSI and liver weight traits were co-localized on LGs 20 and 22.

**Table 4:** Descriptive statistics of LG, including the position, 95% CI, LOD score, PVE, associated *P* value, and specific additive and dominance effects of each QTL linked to every phenotype related to growth

Phenotype*^a^*	LG	Position, cM	95% CI, cM	LOD	% PVE	*P* Value, χ^2^	*P* Value, F	Additive Effect	SE	Dominance Effect	SE	Nearest Marker
Weight T4, g	1	70.5	70-83	4.742	4.477	0.032	0.105	19.696	0.2342	29.897	0.1300	OMM-1195
	3	44.8	20-48	4.945	3.255	0.009	0.046	22.488	0.2365	25.514	0.0845	OMM-5007
	4	43.0	39-47	7.438	5.386	0	0.005	28.711	0.8577	12.158	0.4404	OMM5155i
	5	26.7	21-28	5.267	8.826	0.197	0.352	10.377	0.1783	14.236	0.0952	CA-378164
	9	40.2	39-43	5.137	4.009	0.303	0.468	23.987	0.1054	34.588	0.2607	SSA-0072BSFU
	12	78.4	73-78.4	8.803	6.783	0.203	0.359	3.5396	0.0340	−40.241	0.0346	OMM-5060
	22	17.1	14-19	5.137	5.841	0.087	0.204	44.324	0.2342	5.8255	0.1300	OMM-3015i
	24	11.9	41-94	8.583	6.433	0.162	0.309	8.9338	0.2365	31.112	0.0845	OMM-1201
	27	0.0	0-6	5.858	6.027	0.003	0.02	20.090	0.8577	19.243	0.4404	OMM-5102ii
	31	0.0	0-5	7.293	4.866	0.083	0.199	−4.786	0.1783	12.887	0.0952	C129
	34	0.0	0-12	5.185	5.521	0.142	0.283	9.2407	0.1054	23.328	0.2607	BX319411i
	40	52.4	46-90	5.633	4.745	0.021	0.079	17.955	0.0340	−0.681	0.0346	BX-079862
Specific growth rate, T1–T3, % day^-1^	12	78.4	76-78.4	4.276	4.669	0.036	0.051	−0.016	0.2365	0.2152	0.0845	OMM-5060
	20	0.0	0-37	4.405	3.984	0.041	0.057	0.0519	0.8577	0.1643	0.4404	OMM1210
	22	17.1	0-17	3.227	5.515	0.048	0.056	−0.306	0.1783	−0.089	0.0952	OMM-3015i
	34	0.0	0-5	3.605	4.474	0.042	0.051	−0.166	0.1054	−0.151	0.2607	BX319411i
Specific growth rate, T1–T4, % day^-1^	1	70.5	70-74.5	14.275	4.587	0	0.009	−0.171	0.0340	−0.158	0.0346	OMM-1195
	2	78.2	78-115	8.016	4.119	0	0.022	0.1342	0.2342	0.0340	0.1300	OMY-RGT2TUFii
	3	44.8	10-46	8.509	3.083	0	0.003	−0.121	0.0371	−0.112	0.0481	OMM-5007
	9	40.2	39-42	10.781	5.022	0.028	0.019	−0.175	0.0371	−0.128	0.0370	SSA-0072BSFU
	12	78.4	76-78.4	11.862	4.628	0.003	0.049	−0.039	0.1488	0.1737	0.0532	OMM-5060
	14	45.9	36-47	6.128	9.376	0.039	0.225	0.1685	0.1574	0.0419	0.0829	OMI-30TUFi
	20	0.0	0-38	10.361	3.449	0	0.012	0.0416	0.5659	0.1424	0.2905	OMM1210
	22	17.1	0-17.1	9.466	3.468	0.005	0.08	−0.216	0.1228	−0.079	0.0656	OMM-3015i
	23	13.1	9-25	8.034	5.03	0.027	0.018	0.1479	0.1771	0.1234	0.0891	BHMS-238
	24	11.9	9-13.5	13.409	4.148	0	0.021	−0.016	0.0343	−0.175	0.0334	OMM-1201
	31	42.4	36-42.4	13.392	4.122	0.019	0.015	0.1125	0.3519	0.0904	0.4061	sf004732_01CG
	34	0.0	0-5	12.315	4.175	0	0.02	−0.109	0.0753	−0.085	0.1861	BX319411i
Size T4, mm	3	49.2	45-49.2	11.915	5.067	0.027	0.035	8.904	1.7452	4.320	0.8313	sf000071_02CT
	4	32.7	34-50	13.65	4.618	0.036	0.048	4.422	0.5432	−0.093	0.0803	sf003084_01AG
	12	78.4	52-78.4	14.042	6.148	0.024	0.041	0.3066	1.7646	−1.215	0.6311	OMM-5060
	20	0.0	0-66	12.296	5.012	0.049	0.042	−1.032	0.8970	−0.89	0.2640	OMM1210
	22	20.6	0-20.6	12.262	6.302	0.045	0.038	10.499	4.1333	3.6166	1.4460	sf004055_02CT
	24	1.5	0-6	15.655	4.703	0.047	0.046	6.2293	2.3095	1.7749	1.2464	sf003334_01CT
	26	11.4	8-14	12.8	3.657	0.045	0.043	0.0000	0.094	8.8897	3.7901	sf003018_09CT
	38	28.5	25-29.5	13.278	6.273	0.023	0.031	−1.209	0.8342	−0.576	0.5330	OMM1345
Fulton’s condition factor T4	24	49.3	21-49.3	3.018	4.359	0.036	0.053	3.1857	8.3655	2.0000	2.1284	sf003442_01CG
	38	0.0	0-11	3.57	8.214	0.027	0.035	0.1998	0.4860	0.3300	0.7019	OMY21INRAiii
Liver fresh weight T3, mg	11	0.0	0-11	6.752	9.037	0.006	0.036	−0.893	0.5955	−0.803	0.3896	BX-870052i
	15	52.6	48-52.6	5.698	5.24	0	0.002	−1.024	4.0172	−0.419	2.0618	sf004811_AT
	20	58.1	30-59	9.649	5.99	0	0.001	0.9517	0.3583	1.2504	0.3946	CA-376300i
	22	16.3	12-17	7.517	9.788	0.047	0.053	1.6254	1.2706	0.0679	0.8132	OMM-5147
	24	11.9	8-16	9.996	4.01	0.006	0.037	0.4826	0.0568	1.3452	0.0945	BHMS-238
	34	0.0	0-9	5.595	6.965	0.006	0.039	0.3635	0.3583	0.4824	0.1946	BX319411i
	40	52.4	49-105	9.808	7.119	0.031	0.051	−0.595	1.2706	0.2837	0.0132	SFO-226
Hematocrit T3, % of red blood cells	4	10	5-45	3.337	17.279	0.002	0.002	15.473	4.4098	4.3119	2.2291	OMM-1220
Plasma chloride T3, mmol L^-1^	7	36.6	19-36.6	4.428	5.066	0.045	0.051	0.5712	0.6385	0.2201	0.9368	OMM-5312i
Plasma osmolality T3, mmol kg^-1^	10	42	35-46	3.814	9.257	0.039	0.046	−3.019	0.6014	−4.342	0.4977	SAL5UoG
Plasma glucose T3, mg mL^-1^	10	26	12-62	3.241	11.432	0.016	0.02	14.754	2.3124	−1.468	1.2598	sf004715_AC
Hepatic glycogen T3, mg g liver^-1^	2	40.2	20-77	9.48	4.495	0.029	0.04	9.0189	4.3652	4.8747	2.4390	sf004818_06CT
	36	0.0	0-21.5	5.149	10.576	0.017	0.022	8.9957	1.7665	4.3860	1.7135	sf004038_AG
*ghr* mRNA level T3	40	66	30-95	4.401	12.479	0.032	0.043	−16.17	0.4591	−13.82	0.9399	BX-079862
*igf1* mRNA level T3	3	8.1	5-18	3.391	15.612	0.001	0.002	38.289	1.6790	14.576	0.8526	sf004560_GT
*igf1r* mRNA level T3	3	10.1	5-68	3.178	13.362	0.035	0.047	41.485	0.5037	9.7899	0.3448	OMM5008
Hepato-somatic Index T3	2	112.4	105-117	8.503	5.88	0.019	0.044	0.9172	0.2425	0.4344	0.3797	BX-318599
	3	44.8	12-48	6.31	4.443	0.007	0.027	1.2470	0.1765	0.8431	0.2290	OMM-5007
	4	43.0	32-44	13.266	4.454	0.005	0.045	1.1688	0.2144	1.0091	8.2750	OMM5155i
	5	26.7	23-38	11.077	4.866	0.004	0.09	−0.275	0.1639	0.6595	0.1752	CA-378164
	7	13.7	10-46	4.624	4.387	0	0.003	0.9594	0.2867	−0.025	0.5421	OMM-1263
	20	58.1	20-58.1	15.991	4.948	0.004	0.049	0.9517	0.1440	1.2504	0.1586	CA-376300i
	22	17.1	14-17.1	13.496	6.774	0.002	0.063	2.1143	0.6137	0.2365	0.3277	OMM-3015i
	23	13.1	2-13.1	10.931	4.985	0	0.03	−1.252	0.7850	−0.535	0.3950	BHMS-238
	38	28.5	24-29	14.168	4.007	0.026	0.021	0.3635	0.3590	0.4824	0.2296	OMM1345

Sign (-/+) of the dominance and additive effect indicates to the parent whose allele increases the phenotypic values of the studied trait in the progeny. LG, linkage age; CI, confidence interval; LOD, log_10_ of the odd ratio; PVE, percent variance explained; QTL, quantitative trait loci; SE: standard error of the mean; T4, November; T1, May; T3, August; *ghr*, *growth hormone receptor*; *igf1*, *insulin growth factor-1*; *igf1r*, *insulin growth factor-1 receptor*.

a For each phenotype, the sampling period was added (T1, T3, T4) and the unit of measure is given in parentheses.

For QTL related to growth, the distribution of additive and dominance effects estimated for each of the QTL was relatively large, ranging from −16.17 ± 0.45 (QTL for mRNA *ghr* relative expression) to 44.32 ± 8.36 (g) (mean = 5.58 ± 0.88) for additive effects and from −40.24 ± 0.01 g to 34.58 ± 8.27 g (mean = 3.35 ± 0.66) for dominance effects. Moreover, only one QTL showed an additive effect below 10, and 13 QTL showed additive effects above 10. Two QTL showed dominance effects below 10, and 10 QTL showed dominance effects above 10. For the 63 QTL identified for growth related traits, 30 were associated with an additive gene effect and 20 of these 30 displayed a positive effect. The 33 remaining QTL were associated with a dominance gene effect, and 11 of these 33 QTL displayed a positive effect. Only 12 QTL were linked to SNP markers in contrast with 51 for microsatellite markers. Within these 12 SNP markers, five were identified in coding regions, and two of these were characterized as nonsynonymous changes (Table 6). Seven of these 12 SNP were characterized as transitions, and five were annotated (Table 6). Finally, no significant QTL were detected for five of the 24 (20.8%; 5/24) growth-related phenotypes. These were related to specific growth rate (SGR _T1-T2_), Fulton’s condition factor measured in May (T1) and July (T2), and to level of expression of the *ef1* and *β-actin* genes.

##### Traits related to the stress response:

**Figure 2:**
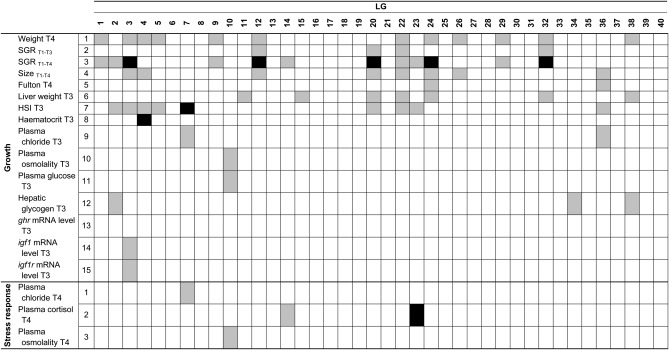
Distribution of QTL related to growth and stress response across the 40 LG identifying the chromosome-wide (gray blocks) and genome-wide significance levels (black blocks). SGR, specific growth rate; HSI, hepatosomatic index; T1, May; T2, July; T3, August; T4, November; *ghr*, *growth hormone receptor*; *igf1*, *insulin growth factor-1*; *igf1r*, *insulin growth factor-1 receptor*.

Four QTL were identified for the three traits related to the stress response: two QTL were related to plasma cortisol, whereas one significant QTL was found for plasma chloride and another for osmolality. The QTL for cortisol level on LG 23 was significant at the genome-wide level, whereas the three others were significant at the chromosome-wide level (QTL for plasma chloride on LG7, QTL for osmolality on LG10, and the other QTL for plasma cortisol on LG 14). The minimal, maximal, and average LOD scores associated with the four QTL were estimated at 3.21, 8.16, and 4.64, respectively (Table 5). The minimal, maximal, and average PVE were estimated at 3.85% (QTL for plasma cortisol on LG7), 31.35% (QTL for the level of cortisol on LG23), and 13.79%, respectively (Table 5). The distribution of additive (from −2.12 μg dL^-1^ ± 0.59 for the plasma osmolality QTL to 0.63 mmol L^-1^ ± 0.49 for the plasma chloride QTL) and dominance (from −3.98 mmol kg^-1^ ± 0.53 for the plasma osmolality QTL to 8.87 μg dL^-1^ ± 2.53 for the plasma cortisol QTL) effects estimated for each of the four QTL related to stress response was relatively weak (Table 5). Of these four QTL, only one was associated with a positive additive gene effect while the three others were associated with a dominance effect (positive for two and negative for one). Among the four QTL, two were linked to SNP markers (sf003382, sf004319) and two to microsatellite markers (SAL5UoG and OMM-5312 were linked to the QTL associated with plasma osmolality and plasma chloride, respectively). Both SNP markers were identified in coding regions; one was characterized as a transition (sf003382), the other as a transversion (sf004319) annotated as a hydroxymethylglutaryl-coA lyase (HMG-CoA, sf004319) (Table 6). Figure 2 displays the QTL identified over all LG for the traits associated with growth and stress response and their respective significance levels.

**Table 5:** Descriptive statistics of LG including the position, 95% CI, LOD score, PVE, associated *P* value, and specific additive, dominance, and interaction effects of each QTL linked to every phenotype related to stress response

Phenotype*^a^*	LG	Position, cM	95% CI, cM	LOD	% PVE	*P* Value, χ^2^	*P* Value, F	Additive Effect	SE	Dominance Effect	SE	Nearest Marker
Plasma cortisol, μg dL^-1^	14	68.5	27-68.5	3.217	3.854	0.013	0.015	0.0056	0.0091	8.8723	2.5333	sf003382_AG
	23	0.1	0-16	8.161	31.35	0	0	0.0000	0.0098	0.1119	0.0168	sf004319_GT
Plasma osmolality, mmol kg^-1^	10	42	37-47	3.667	11.232	0.039	0.046	−2.123	0.5945	−3.9858	0.5363	SAL5UoG
Plasma chloride, mmol L^-1^	7	36.6	17-38	3.532	8.734	0.045	0.049	0.6324	0.4987	0.2201	0.9856	OMM-5312i

LG, linkage age; CI, confidence interval; LOD, log_10_ of the odd ratio; PVE, percent variance explained; QTL, quantitative trait loci; SE: standard error of the mean.

a All the phenotypes related to the stress response were measured at T4 (November) and the unit of measure is given in parentheses.

**Table 6:** Detailed information related to the SNP markers linked to QTL associated with growth and stress response

Phenotype	SNP Name	Accession Number	GI	Variation	Ts/Tv	C/NC	S/NS	LG	Available Annotation
Growth									
Size	sf000071			A/C	Tv	NC	−	3	−
Size	sf003018			A/G	Ts	NC	−	26	−
Size	sf003084			A/G	Ts	C	−	4	−
Size	sf003334			A/G	Ts	NC	S	24	−
Fulton index	sf003442			A/G	Ts	C	S	24	−
Hepatic glycogen	sf004038	NM_001165151	GI:259089170	A/G	Ts	NC	−	34	Ubiquitin-conjugating enzyme E2W
Size	sf004055	XP_001921123	GI:189530039	A/T	Tv	C	NS	22	Neural-cadherin-like
* igf1* mRNA level	sf004560			G/T	Tv	NC	−	3	−
Plasma glucose	sf004715			C/T	Ts	C	S	10	−
SGR	sf004732	AB258536	GI:118596560	C/T	Ts	NC	−	29	Onmy-LDA gene for MHC class I antigen
Liver fresh weight	sf004811	EU025709	GI:158702304	A/T	Tv	C	NS	15	−
Hepatic glycogen	sf004818	EU481821	GI:171474994	A/C	Tv	NC	−	2	Formin-binding protein 1
Stress response									
Plasma cortisol	sf003382			A/G	Ts	C	NS	14	−
Plasma cortisol	sf004319	BT058994	GI:223647897	G/T	Tv	C	S	23	Hydroxymethylglutaryl-CoA lyase, mitochondrial precursor putative mRNA

SNP, single nucleotide polymorphism; QTL, quantitative trait loci; GI, GenInfo identifiers; Ts, transition; Tv, transversion; C, coding region; NC, noncoding region; S, synonymous; NS, nonsynonymous; LG, linkage group.

## Discussion

### Growth-related QTL

This study allowed the identification of QTL for a total of 27 phenotypes related to two physiological traits of economical importance in *S. fontinalis*, one of the most economically important salmonid species for freshwater aquaculture. First, this allowed the identification of 63 growth-related QTL. The low number of QTL identified genome-wide (only eight) compared with the high number identified chromosome-wide (55) is in accordance with previous studies in salmonids in which authors identified QTL related to body weight and condition factor in Arctic charr *S. alpinus* (Kuttner *et al.* 2011) or QTL related to growth in domestic and wild rainbow trout *Onchorynchus mykiss* (Wringe *et al.* 2010). The limited number of molecular markers and sample size used in our study may partly explain these observations because these factors tend to overestimate the magnitude of large-effect QTL and hide the numerous small-effect QTL (Mackay *et al.* 2009). Note, however, Lander and Kruglyak (1995) strongly recommend the use of a genome-wide significance threshold, regardless of the density of the map used, to further deploy many additional markers in regions that show signs of a segregating QTL after an initial sparse map search (Van Ooijen 1999). Similarly, although the low number of QTL identified per trait may indicate that the expression of these traits is under the control of one major or a limited number of genes, we cannot refute the possibility that it may also reflect the limited lack of power of the study. Namely, the limited number of progeny may influence QTL detection experiments by underestimating the QTL number and overestimating their respective effects (Beavis 1998; Xu 2003). Moreover, the fact that the number of detected QTL varied substantially depending on traits suggests that power limitation is not the only factor explaining the detection of one or very few QTL for some of these, especially for polygenic traits (*i.e.*, genetic architecture) for which, individual locus contribution (*i.e.*, distributions of effects) is relatively low and remain difficult to detect.

The 63 QTL associated with the growth-related traits detected in this study is comparable with the 64 and 31 growth-related QTL identified by Kuttner *et al.* (2011) and Wringe *et al.* (2010) in Arctic charr and rainbow trout, respectively, on the basis of maps comprising 106 and 137 markers, respectively. Admittedly, some assumptions made for the analysis may have contributed to overestimates in the number of detected QTL. Namely, we assumed that each QTL corresponded to an independent repeated observation. This assumption was a simplification because some QTL were found at very similar chromosomal positions (*i.e.*, colocalization) and thus might be the same (*i.e.*, growth-related QTL). In addition, some traits are correlated and hence QTL for two correlated traits at a similar chromosomal position might in fact be a single pleiotropic QTL (*i.e.*, because of the effect of a single gene on multiple phenotypic traits). Jansen *et al.* (1995) proposed statistical methods to test for the pleiotropic effect of genes. However, the application of these methods remains challenging when using low- to medium-density genetic linkage map, as was the case here and in the majority of QTL analysis in salmonids thus far. This is also particularly problematic for salmonids in general, given the lower recombination rate in males that leads to a restricted resolution of the male genetic map (Moghadam *et al.* 2007b). Nevertheless, estimations of QTL positions at the genome scale show relatively large 95% confidence interval (95% CI) (Visscher *et al.* 1996) and hence are not estimated with enough precision to claim that the QTL positions are a single pleiotropic QTL (Bennewitz and Meuwissen 2010). The most probable locations of the 63 growth-related QTL were identified with varying degrees of accuracy, with minimal, maximal, average, and median 95% CI of 2.4, 66, 19.7, and 12 cM, respectively. Again, these values are comparable with those previously reported in Arctic charr (Kuttner *et al.* 2011) and rainbow trout (Wringe *et al.* 2010).

The number of QTL detected and their associated PVE for each trait also provide further insight into the genetic architecture underlying the variance of the phenotypic traits studied. Phenotypes such as weight, length, SGR, HSI, and liver fresh weight typically characterizing growth traits generally showed numerous QTL (ranging from four to nine) associated with small PVE (ranging from 3.07 to 9.37). This finding suggests that these phenotypes are under the control of a large number of genes of relatively small effects. On the contrary, hematocrit, plasma chloride, plasma glucose, plasma osmolality, hepatic glycogen, and mRNA levels of *ghr*, *igf1*, and *igf1r* showed few QTL (<2) with generally larger PVE (up to 17.27%). This finding suggests that these phenotypes are under the control of one or two major genes of larger effects. In total, however, only six QTL (linked to hematocrit, plasma glucose, hepatic glycogen, and mRNA levels of *ghr*, *igf1*, and *igf1r*) showed PVE over the 10% threshold and can be characterized as large-effect QTL.

Although correlations between microsatellite markers and the variation of traits related to growth and reproduction were also investigated in other salmonids, such as rainbow trout (Wringe *et al.* 2010) and Arctic charr (Kuttner *et al.* 2011), it was not possible to compare the colocalization of these previously identified QTL with those identified in the present study because of the low number of microsatellite marker pairs shared between these studies.

### Identification of SNP markers linked to growth-related QTL

For each QTL position, the closest underlying molecular marker was identified. Of the 63 growth-related QTL, only 12 were linked to a SNP marker, whereas the 51 others were linked to a microsatellite marker. Moreover, only five of these 12 were identified in coding regions; two of these five were characterized as nonsynonymous changes, and five of the 12 (41.6%; 5/12) had a significant annotation. The seven remaining SNP were identified in UTR or noncoding regions. Thus, direct assumptions of the role played by the genes linked to QTL in the genetic architecture of the phenotypic traits remain difficult, especially since the molecular function of these five genes was not directly related to growth, considering their annotation. We thus suggest that these SNP markers are in linkage disequilibrium with the actual “causative locus.” For instance, previous results obtained for another salmonid, the lake whitefish *Coregonus clupeaformis*, showed that expression QTL were located within a hotspot of recombination where a putative *trans*-regulating element was identified (Zinc finger protein 35; reviewed in Bernatchez *et al.* 2010).

Of the 63 growth-related QTL in this study, similar proportions were associated with dominance (52.4%; 33/63) or additive (47.6%; 30/63) effect. This measure gives insight about the genetic architecture of the trait, *i.e.*, about the gene action associated with the QTL and the effect of the underlying alleles. Sign (−/+) of the dominance or additive effect, at each locus, corresponds to the parent whose allele increases the phenotypic values of the studied trait in the progeny (Lynch and Walsh 1998).

### Correlations between phenotypes related to growth performance

A positive correlation was observed between glucose plasma level and the expression of genes related to growth performance (*ghr*, *igf1*, *igf1r*). It is known that glucose availability and utilization are regulated by endocrine factors involved in appetite, growth, and energy homeostasis, and that glucose exerts a feedback loop on these endocrine pathways. In particular, glucose plays a role in the GH/IGF1 growth axis in all vertebrates, including fish (Pérez-Sánchez *et al.* 1994; Banos *et al.* 1998; Riley *et al.* 2009). In particular, our results are in accordance with those of Riley *et al.* (2009), who demonstrated that a dietary glucose load significantly increased the mRNA level of two genes coding for two growth hormone receptors (*ghr1* and *ghr2*) in tilapia *Oreochromis mossambicus*. Moreover, Banos *et al.* (1998) showed that rainbow trout fed high-carbohydrate diets had an increased liver *Igf1r* number and specific binding. Contrary to our results, however, Riley *et al.* (2009) reported a stable liver *igf1* mRNA level as well as a reduced plasma *igf1* level in tilapia with a plasma glucose load. This finding could indicate that the relationship between the plasma glucose level and the GH/IGF1 axis may differ according to the species considered. We also measured a positive correlation between glucose plasma level and mRNA levels of *ef1* and *β-actin*. *β-actin* is involved in cell motility, structure, and integrity, whereas *ef1* has been shown to play a central role in protein synthesis in teleosts (Jurss *et al.* 1992). Our results thus suggest the essential role of glucose as energy supplier to the cell to perform these vital functions in *S. fontinalis*.

### QTL related to stress response

The present study identified four QTL associated with the three phenotypes related to stress response and localized on four different LGs. Two QTL were identified for the level of plasma cortisol (LG 14 and 23; chromosome- and genome-wide level of significance, respectively), whereas only one QTL was identified for plasma osmolality (LG 10; chromosome-wide level of significance) and one for plasma chloride (LG 7; chromosome-wide level of significance). The PVE estimation of 31.35% revealed a major effect for the QTL associated with plasma cortisol (LG 23). The PVE was weaker for another QTL associated with plasma cortisol (LG 14; PVE = 3.85%), as well as QTL associated with plasma osmolality (LG 10; PVE = 11.23%), and plasma chloride (LG 7; PVE = 8.73%). The small number of QTL identified for these traits combined with general high PVE values suggests that the genetic architecture of these four phenotypes is under the control of a limited number of genes or a major gene effect. Low number of QTL related to the cortisol stress response has previously been reported in other vertebrates (pigs: Ousova *et al.* 2004; rodents: Garlow *et al.* 2005 and Llamas *et al.* 2005; and poultry: Beaumont *et al.* 2005). Moreover, Vallejo *et al.* (2009) and Quillet *et al.* (2010) reported a limited number of major genes affecting plasma cortisol levels after a crowding stress in rainbow trout. Again, because QTL analyses are biased toward the identification of large QTL effects, the power of our analysis (*n* = 171 individuals) may be insufficient to detect low to moderate QTL effects influencing these traits. However, this cannot in itself explain the contrast observed between these QTL and those for phenotypic traits related to growth for which a larger number of small-effect QTL was revealed.

In the present study, both QTL associated with plasma cortisol were colocalized with two growth-related QTL (SGR located on LG 14 and 23), suggesting a genetic correlation between stress response and growth performance. This finding is in accordance with Drew *et al.* (2007), who detected one QTL associated with juvenile rainbow trout body mass overlapping with a QTL for cortisol levels, indicating a putative genetic link between these two traits. Moreover, these authors reported additive effects of these two overlapping QTL, suggesting a positive relationship between cortisol levels and growth rate. However, this finding is in contrast with Fevolden *et al.* (2002), who previously reported a negative correlation between stress response and growth performance in rainbow trout. However, this statement should be interpreted cautiously when comparing both studies, as fish were younger in Fevolden *et al.* (2002) and were not subjected to any domestication process that may alter stress response and growth. Taken together, our results and previous ones suggest that the cortisol response after a stress exposure may be under variable genetic control depending on species. This also demonstrates that stress response measured through plasma cortisol is a highly complex trait and may influence a range of underlying physiological mechanisms. In the case of brook charr however, it seems clear that stress responses are under strong genetic control given recently published high heritability estimates (*h*^2^ = 0.60 ± 0.20; *h^2^* = 0.61 ± 0.20) for plasma cortisol and plasma glucose, respectively (Crespel *et al.* 2011). The identification of QTL related to the same phenotypic traits supports the strong potential role of these QTL for genetic improvement of the stress response in brook charr and possibly other salmonid species.

### Identification of SNP markers linked to identified QTL related to stress response

For each QTL position, the closest underlying molecular marker has been identified. Within the four stress-related QTL, two were linked to an SNP marker (sf003382 and sf004319 linked to plasma cortisol on LG 14 and LG 23, respectively), whereas the two others were linked to SSR markers (SAL5UoG and OMM-5312i linked to plasma osmolality and plasma chloride on LG 10 and LG 7, respectively). For the two QTL related to plasma cortisol, the closest linked SNP markers were both identified in a coding region but were characterized as a nonsynonymous transition in one case (sf003382) and as a silent transversion in the other (sf004319). SNP sf004319 was significantly annotated as a precursor of the mitochondrial HMG-CoA (accession number [GenBank:BT058994]). This gene catalyses the transformation of HMG-CoA into acetyl-CoA and acetoacetate. In vertebrates, HMG-CoA is a mitochondrial and paroxysmal enzyme that is involved in ketogenesis and in leucine catabolism (Mitchell *et al.* 1993). As was the case for growth-related QTL that were linked to SNP markers, direct assumptions of the role played by these two SNPs linked to stress-related QTL remain difficult, especially since the molecular function of the only annotated marker was not directly related to stress response. Here again, we suggest that these SNP markers are in linkage disequilibrium with the causative locus (or transcription factors). As such, our results should be perceived as representing a necessary first step toward the identification of the genes underlying the genetic architecture of stress response in fish and of growth performance in *S. fontinalis*. The identification of actual causative locus will require the availability of intensive genetic information and genomic tools, such as dense, gene-rich linkage maps and whole-genome sequences, that are currently in development in salmonids (Davidson *et al.* 2010).

### Conclusion

For the first time in brook charr, and to our knowledge only the second time in the genus *Salvelinus*, this study examined the association between molecular markers and the variance of numerous traits related to growth performance and stress response. Our results can be divided into two groups. The first group may be composed of growth performance-related QTL that are numerous per trait (>2; size, weight, SGR, HSI, liver fresh weight, Fulton index) and display small-to-moderate effects. This suggests that these growth-related traits, as expected for such a complex trait, are under the control of a large number of genes (pleiotropic genes). The second group may be composed of QTL that are less numerous per trait (< 2) and display larger effects, suggesting that these QTL are under the control of a limited number of genes of more important effect. These less numerous QTL were mainly identified for some growth-related traits (hematocrit, plasma chloride, plasma glucose, plasma osmolality, hepatic glycogen and mRNA levels of *igf1*, *igf1r*, *gh*, *ef1*, and *β-actine*) and for all the traits related to stress response (plasma chloride, plasma cortisol, and plasma osmolality). Colocalization of QTL associated with plasma cortisol and with SGR has been identified for the first time in *S. fontinalis*. Admittedly, the identification of genes underlying the genetic architecture of traits of interest, such as growth or stress response, will require improvement in a subsequent map generation. This will require an ever-increasing amount of genetic information and research tools to increase the density of linkage maps. Linkage map improvement will also benefit from the increasing availability of whole-genome sequences in fish that are currently in development in salmonids (Davidson *et al.* 2010) and the more widespread use of the latest NGS techniques for development in nonmodel species in general (Amores *et al.* 2011; Rowe *et al.* 2011).
